# Transcranial direct current stimulation and attention skills in burnout patients: a randomized blinded sham-controlled pilot study

**DOI:** 10.12688/f1000research.21831.2

**Published:** 2020-07-31

**Authors:** Pia Van Noppen, Kim van Dun, Siel Depestele, Stefanie Verstraelen, Raf Meesen, Mario Manto

**Affiliations:** 1 DIADIS BVBA, Oud-Turnhout, Antwerpen, 2360, Belgium; 2 Neuroplasticity and Movement Control Research Group, Rehabilitation Research Institute (REVAL), UHasselt, Diepenbeek, Limburg, 2590, Belgium; 3 Clinical and Experimental Neurolinguistics (CLIN), Vrije Universiteit Brussel (VUB), Brussels, Belgium; 4 Service de Neurologie, CHU-Charleroi, Charleroi, Henegouwen, 6041, Belgium; 5 Service de Neurosciences, Université de Mons, Mons, Henegouwen, 7000, Belgium

**Keywords:** burnout, direct current stimulation, attention, working memory, prefrontal cortex

## Abstract

**Background: **Burnout is characterized by deficiencies in attention and several components of the working memory. It has been shown that cognitive behavioral therapy can have a positive effect on burnout and depressive symptoms, however, the lingering effects of impaired attention and executive functions are the most frustrating. We hypothesized that anodal transcranial direct current stimulation (atDCS) over the left dorsolateral prefrontal cortex (DLPFC) can improve the executive control of attention and possibly several other components of working memory in patients with burnout.

**Methods: **This was a randomized double-blind sham-controlled pilot study with two groups. Patients with burnout received three weeks of daily sessions (15 sessions in total) of atDCS or sham stimulation in addition to three weekly sessions of standard behavioral therapy. The primary outcome measure was attention and the central executive of the working memory. Secondary, the effect of atDCS was measured on other components of working memory, on burnout and depression scores, and on quality of life (QoL).

**Results: **We enrolled and randomly assigned 16 patients to a sham or real stimulation group, 15 (7 sham, 8 real) were included in the analysis. atDCS had a significant impact on attention. Post-hoc comparisons also revealed a trend towards more improvement after real tDCS for inhibition and shifting, updating and control, and encoding. Both groups improved on burnout and depression scores.

**Conclusion:** These data provide preliminary evidence for the value of atDCS over the left DLPFC in rehabilitating attention deficits, and possibly also central executive and encoding deficits, in burnout. However, the current study has some limitations, including the sample size and heterogeneous patient population. More elaborate studies are needed to elucidate the specific impact of atDCS over the left DLPFC on burnout.

**Trial registration: **ISRCTN.com ( ISRCTN94275121) 17/11/19

## Abbreviations

**atDCS** anodal transcranial Direct Current Stimulation

**BDI** Beck’s Depression Inventory

**BNT** Boston Naming Test

**DSM-V** 5 ^th^ edition of the Diagnostic and Statistical Manual for Mental Disorders

**ICD-10** 10 ^th^ edition of the International Statistical Classification of Diseases and Related Health Problems

**MBS** Maslach Burnout Scale

**NMDA** N-Methyl-D-Aspartate

**RBANS** Repeatable Battery for the Assessment of Neuropsychological Status

**SD** Standard Deviation

**tDCS** transcranial Direct Current Stimulation

**TMT** Trail Making Test

**WCST** Wisconsin Card Sorting Test

**QoL** McGill Quality of Life Questionnaire

## Introduction

The percentage of employees experiencing burnout is dramatically increasing in Europe ( Eurofound, 2018), which has a significant socio-economic impact. Burnout consists of three components: (1) exhaustion at the physical level (energy loss, fatigue, weakness, physical and psychosomatic complaints), the mental level (negative behavior towards oneself, work, or life in general), or the emotional level (feelings of being trapped in a situation, helplessness, or hopelessness); (2) depersonalization or alienation towards the actual work, towards patients or pupils, etc. ( Demerouti *et al*., 2001; Schaufeli & Enzmann, 1998); (3) and reduced professional performance, which can be attributed to depersonalization and alienation ( Demerouti *et al*., 2001). Since the beginning of 2011, burnout has been added to the 10 ^th^ edition of the International Statistical Classification of Diseases and Related Health Problems (ICD-10: Z73.0: Word Health Organization, 2011), which describes burnout as a 'state of vital exhaustion'. The 5 ^th^ edition of the Diagnostic and Statistical Manual for Mental Disorders (DSM-V: American Psychiatric Association, 2013), on the other hand, categorizes burnout under 'somatic symptoms and related disorders'.

**Figure 1.**
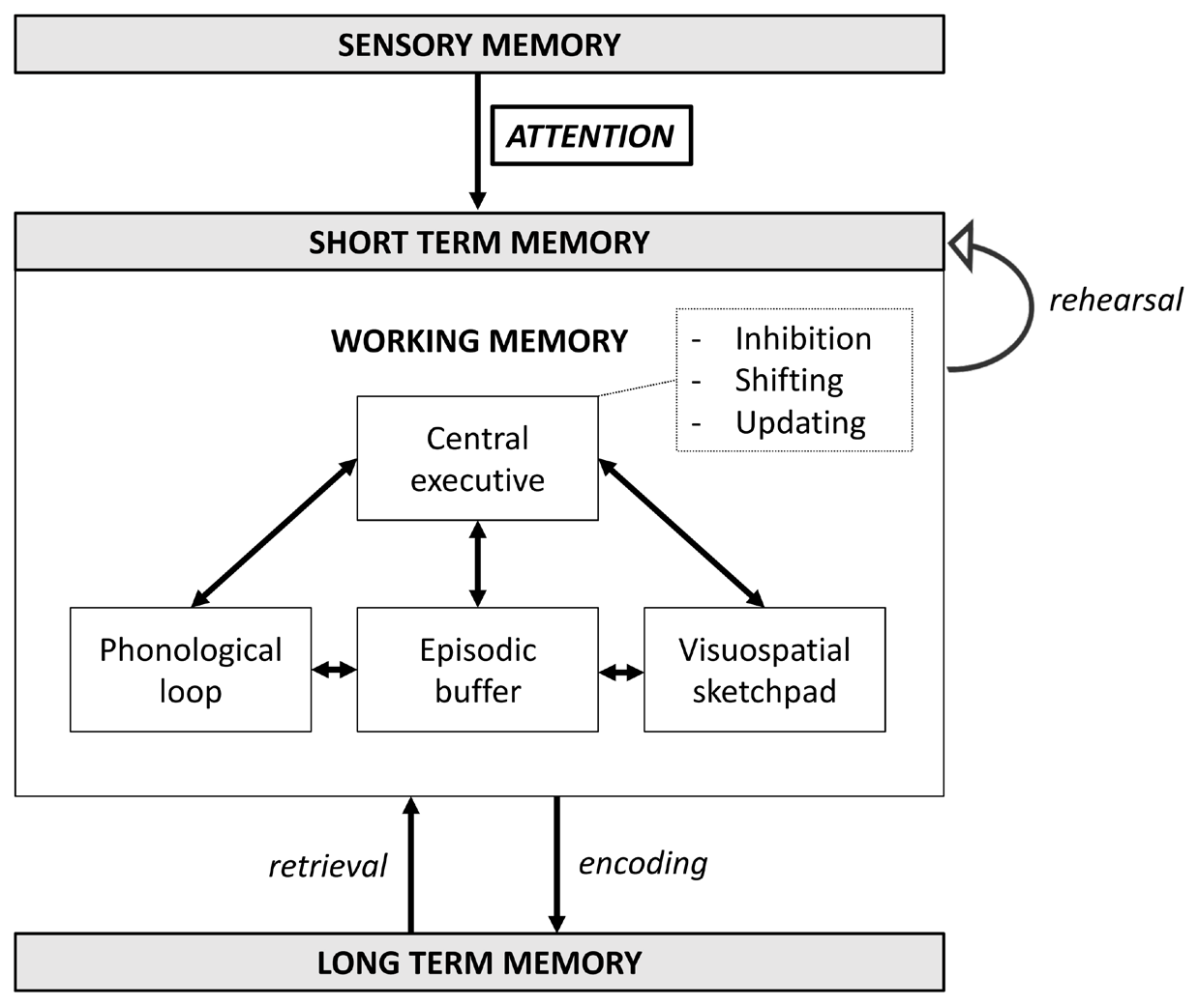
Memory model with the different components of the working memory and the interactions between the short-term and the long-term memory, based on ( Baddeley, 2003).

Patients with burnout are impaired in one or more of the four components of working memory, i.e. the central executive, the phonological loop, the visuospatial sketchpad and/or the episodic buffer (see Figure 1) ( Baddeley, 2000; Deligkaris *et al*., 2014). The working memory, or the short-term memory, refers to a limited-capacity cognitive system that allows the temporary storage and manipulation of information from different modalities, provided by the sensory memory, that are necessary for complex tasks. (1) The phonological loop is responsible for encoding language in the long-term memory and for short-term retention of phonological information through repetition ( Baddeley *et al*., 1998). (2) The visuospatial sketchpad temporarily stores visual and spatial information. (3) The episodic buffer temporarily stores and integrates information from the other components, and links information to time and space to make storage and invocation easier ( Baddeley, 2000). These three components are controlled by the fourth component, i.e. (4) the central executive, which ensures that targeted actions can be taken by guiding attention towards relevant information in the sensory memory ( Baddeley, 1996). The central executive operates by (1) inhibition, i.e. the suppression of dominant, automatic answers, and the resistance to interference caused by distractors; (2) shifting, which refers to the possibility to switch cognitively between various tasks, mental states, or operations; and (3) updating of the working memory ( Miyake *et al*., 2000).

The working memory does not only monitor and direct attention, it is also responsible for the storage of information in the long-term memory (encoding) and recall of information from that same memory (retrieval) ( Baddeley, 1996; Baddeley & Sala, 1996).

Based on this model, deficits of executive functions and attention could be attributed to dysfunction of the central executive component ( Baddeley, 1996). Accordingly, impairment of nonverbal memory deficits could be associated with the visuospatial sketchpad ( Papagno, 2002), verbal memory deficits could be connected to the phonological loop ( Vallar & Baddeley, 1984), and episodic (long-term) memory disruption could be attributed to dysfunction of the episodic buffer ( Quinette *et al*., 2006). However, not all components of the working memory model are equally affected in burnout. A recent meta-analysis stated that burnout primarily affects attention, vigilance (i.e. sustained attention), and the central executive, more specifically memory updating and monitoring ( Riedrich *et al*., 2017).

Transcranial direct current stimulation (tDCS) is a non-invasive neurostimulation technique that modulates cortical excitability to enhance brain function by means of a low electrical current applied over the skull ( Brunoni *et al*., 2012; Nitsche & Paulus, 2011). tDCS is increasingly used in the treatmentof motor, cognitive, and affective symptoms in different patient populations, both in neurological (e.g. Alzheimer’s disease; Flöel, 2014), and psychiatric disorders (e.g. major depressive disorder Nitsche *et al.*, 2009) ( Brunoni *et al*., 2012). The therapeutic potential of tDCS is gaining interest. In a double-blind sham-controlled trial consisting of three weeks (15 sessions) of active or sham anodal tDCS (atDCS) (2mA) over the left dorsolateral prefrontal cortex (DLPFC), Loo *et al.* confirmed the antidepressant efficacy of atDCS in patients with depression. In addition, mood, attention skills, and working memory also significantly improved after active tDCS treatment ( Loo *et al*., 2012). Moreover, a recent study by Miler, Meron, Baldwin, and Garner showed that a single session of DLPFC stimulation can improve executive control of attention in healthy adults ( Miler *et al*., 2018). However, to induce a longer-lasting effect, repeated sessions are advised and it has already been shown that this can have a cumulative effect which is associated with greater magnitude and longer duration of the behavioral effects ( Brunoni *et al*., 2012).

One of the mechanisms that might be responsible for the cognitive problems in burnout patients is a dopaminergic dysfunction in the prefrontal cortex. It has been shown that dopamine in the prefrontal cortex plays a critical role in working memory and cognitive control ( Polizzotto *et al*., 2020; Cools & D’Esposito, 2011) and that (chronic) stress can have a deteriorating effect on the dopaminergic system in this area ( Mizoguchi *et al*., 2000). tDCS has been known to interact with dopaminergic systems ( Polizzotto *et al*., 2020) and therefore tDCS over the DLPFC might be able to restore dopaminergic prefrontal cortex function.

The effects of tDCS have not yet been extensively evaluated in burnout patients. Some studies have used tDCS in stress-related patient populations, such as professional nurses ( Stanton *et al*., 2015) or post-traumatic stress disorder ( Saunders *et al*., 2015), however, to our knowledge, our study is the first to use tDCS in a burnout population. 

Studies have shown that burnout patients are primarily impaired in attention and the central executive ( Riedrich *et al*., 2017). We tested the hypothesis that atDCS over the left DLPFC could improve the general well-being of recovering burnout patients by boosting the recovery of the executive control of attention. Since this is the first study using tDCS in the rehabilitation of burnout patients, other components of the working memory were also measured to monitor the impact of burnout and the effect of atDCS on these components.

## Methods

### Patients

Patients were recruited between January 2015 and December 2017 via a treatment center in Belgium specialized in the diagnosis and treatment of burnout (DIADIS NV, Oud-Turnhout). The definition of ( Brenninkmeijer *et al*., 2001) was used to identify burnout patients, and a score of > 4 on the Dutch version of the Maslach Burnout Scale (MBS: Maslach-Pines, 2005) was considered an inclusion criterium. Patients with 1) excessive drug or alcohol use, 2) epilepsy, 3) depression, 4) bipolar syndrome, 5) chronic fatigue syndrome or any other history of psychiatric or neurological disorders, 6) implanted neurostimulator or pace-maker, 7) drugs interacting directly with the NMDA receptors, or 8) pregnancy were excluded. When new patients were diagnosed with burnout in the treatment center, they were asked whether they wanted to participate in the study. Included patients were pseudo-randomly assigned to a real atDCS or sham tDCS group using a pre-defined allocation code file in excel (to make sure that both groups were of equal size). Initially, 20 participants were targeted (10 per tDCS group) as a pilot study. This number was primarily based on practical issues, such as the average number of burnout patients that were treated every year at the treatment center, and the time the treating psychologist could devote to the study. All assessments were performed by the sole psychologist of the treatment center (PVN). 

This study was approved by the ethical committee CME of the Vrije Universiteit Brussels (VUB) (B.U.N. 143201422009). All patients signed an informed consent. The trial was retrospectively registered at ISRCTN.com on 17/11/19 ( ISRCTN94275121), since clinical trial registration was not explicitly required by the advising ethical committee for trials with an experimental device at the start of the trial. All protocol and trial details are available from the registration page.

### Pretesting

After inclusion, baseline measures were taken to evaluate burnout, depression, quality of life, attention, and different components of the working memory. Burnout, depression, and overall quality of life were assessed by the MBS, the Beck’s Depression Inventory (BDI: Van der Does, 2002), and Question A of the Dutch version of the McGill Quality of Life Questionnaire (QoL: Cohen *et al*., 1997); translated by Kenniscentra Palliatieve Zorg) respectively.

Attention was measured by the Repeatable Battery for the Assessment of Neuropsychological Status (RBANS: Randolph, 1998) Attention Index, and vigilance by the s-score of the D2 test (variability in processing speed).

The central executive of the working memory was evaluated with the following tests. Inhibition and shifting were assessed with Card III of the Stroop Color-Word test ( Golden, 1978), the Trail Making Test part B (TMT: Reitan, 1958) and the Wisconsin Card sorting test (WCST: Heaton *et al*., 1993). Processing speed, i.e., updating and control, was assessed by the TMT part A, Cards I and II of the Stroop Color-Word test, and the D2 test (G _z_: total number of tokens scanned; F%: error percentage relative to G _z_; G _z _– F: number of correctly identified tokens) ( Brickenkamp, 1962).

As regards to the other components of the working memory: the phonological loop was tested by the Language Index of the RBANS, the Boston naming test (BNT: Kaplan *et al*., 1983; Flemish version BNT: Mariën *et al.*, 1998) and semantic fluency tasks (naming as many animals, vegetables, means of transportation and clothes as possible within one minute). To determine the percentile of semantic fluency, Dutch non-published age-, gender-, and education-related norms were used (These data were obtained by master students in Linguistics at the VUB of 200 healthy participants in Belgium of varying age, gender, education, and geographic location and are available as extended data ( van Dun, 2020)). These data were used to calculate the z-scores that were then converted to percentiles. The visuospatial sketchpad was assessed using the Raven’s progressive matrices ( Raven, 1965), and the Visuospatial Index of the RBANS. Encoding was evaluated with the Immediate Memory Index and retrieval with the Recent Memory Index of the RBANS.

A categorized overview of the different tests is presented in Table 1.

**Table 1.** Overview of the different tests for measuring relevant impaired functions accompanying burnout.

Category		Test	Mean (SD) or Maximum score or Type of score
**Burnout**		MBS	Max 7
**Depression**		BDI	Max 63
**Quality of Life**		QoL	Max 10
**Attention**		RBANS Attention Index	100 (15)
**Vigilance**		D2 (s-score)	Pct.
**Working memory**			
Central executive	*Inhibition and* *shifting*	Stroop III TMT B WCST	Pct. Pct. #Categories
	*Processing speed* *(updating and* *control)*	TMT A Stroop I and II D2 (G _z_, F%, G _z_ – F)	Pct. Pct. Pct.
Phonological loop		RBANS Language Index	100 (15)
		BNT	SS
		Semantic fluency tasks	Pct.
Visuospatial sketchpad		Raven	100 (15)
		RBANS Visuospatial Index	100 (15)
**Encoding**		RBANS Immediate Memory Index	100 (15)
**Retrieval**		RBANS Recent Memory Index	100 (15)

[i] **Legend**: MBS = Maslach Burnout Scale; BDI = Beck’s Depression Inventory; QoL = McGill Quality of Life Questionnaire; RBANS = Repeatable Battery for the Assessment of Neuropsychological Status; Stroop = Stroop Color-Word test; TMT = TrailMaking Test; WCST = Wisconsin Card Sorting Test; BNT = Boston Naming Test; Raven = Raven’s progressive matrices; SS = Standard Score; Pct. = Percentile.

After treatment, all tests were repeated to evaluate the impact of atDCS. Therapy always started on a Monday, and re-evaluation was completed the first Monday after the final atDCS session.

### Primary and secondary outcome measures

The primary outcome measure was attention. Secondary outcome measures were general measures (burnout, depression, and quality of life), and other components of the working memory (central executive, phonological loop, visuospatial sketchpad, encoding and retrieval).

### Treatment

All patients received the standard behavioral therapy consisting of one session a week (for 3 weeks) focusing on 1) psycho-education and relaxation, 2) reducing mental overload, 3) defining and working to personal goals, 4) relapse prevention. 1) In the first session, the stress mechanism was explained, together with the characteristics that belong to it. Breathing exercises were taught to the patient through heart rhythm coherence, using EmWave2 software to visually guide the patients. 2) To reduce the mental overload, ‘don’t worry’-techniques were explained. Patients were advised to write down their worries and not get distracted by them continuously. Via cognitive behavioral therapy, using the ABCDE model ( Ellis *et al*., 1997), they were taught to translate negative into positive thoughts. 3) During therapy, the patient’s life goals in different domains (e.g. work, personal relations, education, parenthood, friends, physical well-being, …) were established together with the therapist. In dialogue, priorities were established and possible (mental) barriers were discussed. This discussion primarily focused on rebalancing the different domains in the patient’s life. 4) Lastly, the therapy focused on reintegration on the work floor. Bad habits were identified and strategies were discussed to prevent the patients from falling back into these habits.

None of the patients had received psychotherapy before inclusion in this study.

**Figure 2.**
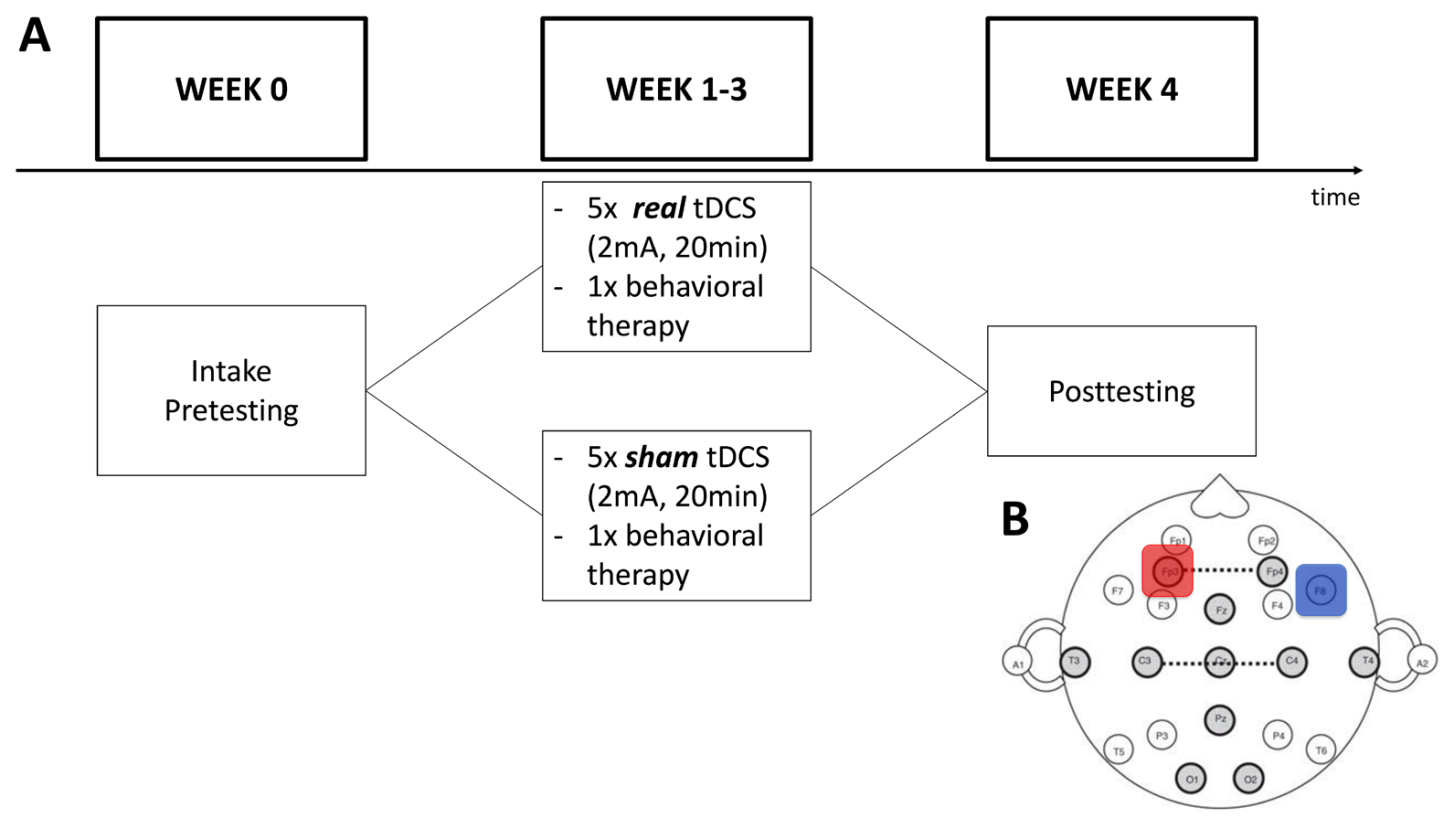
**A**. Visualization of the protocol, and **B**. electrode placement of the tDCS protocol.

In addition, patients received daily sessions of 2mA atDCS (TCT Research Limited, Hong Kong) over the left DLPFC (AF3 on the international 10/20 EEG system) (electrode size: 5x5cm ^2^) and the reference electrode (5x7cm ^2^) over the lateral aspect of the contralateral orbit (F8), as described in ( Loo *et al*., 2012). The carbon electrodes were covered in sponges soaked in saline solution (0.9% NaCl) to improve conductivity. These were placed over the scalp using neoprene straps. Since these can absorb the saline solution, two different straps were used for both electrodes to avoid creating bridges, and throughout the sessions the absorption was monitored so that it would not spread beyond the surface of the electrodes. In the real tDCS group, stimulation lasted for 20min with a gradual ramp up over 30s. This resulted in a maximal current density of 0.08mA/cm ^2^ and a total charge of 0.096C/cm ^2^ per session. Impedance was continuously monitored during stimulation to stay below 10kOhm and was automatically disrupted for safety when it went above 15kOhm. During sham stimulation, the current was ramped up over 30s to 2mA after which it was immediately ramped down to simulate the cutaneous sensation of tDCS in the sham group. No therapy was given during stimulation. This resulted in 15 sessions in total (3 weeks, 5x/week). One group received real tDCS, the other received sham tDCS. The tDCS device was programmed by the therapist, but the patients did not know which type of tDCS they received. All test results were coded to blind the researcher who performed the analyses and data was unblinded only after the analyses were done. The protocol and electrode placement are illustrated in Figure 2.

All therapy and tDCS sessions were performed at the treatment center DIADIS NV in Oud-Turnhout, where the patients were recruited, by the same psychologist and co-author Pia Van Noppen.

### Statistical analyses

Means and standard deviations (SDs) were reported to give a general overview of the results. An independent samples t-test was used to compare mean age between groups.

 A full-factorial 2 (tDCS: sham, real) x 2 (time: pre, post) fixed effects linear mixed model with subject as a random effect was used to compare the results on all test measures between the sham and real tDCS groups before and after treatment. Normality and homoscedasticity of the residual data were checked via a normal quantile plot and residual plot, respectively. If model assumptions were violated, the outcome variable was transformed using the Box-Cox procedure ( Box & Cox, 1964), as implemented in the MASS package in R version 7.3-51.4. Tukey HSD post-hoc pairwise comparisons were used to compare baseline scores between groups and to explore possible interaction effects. The level of significance was set at α = 0.05. All statistical analyses and figures were generated using the statistical software R version 3.6.0.

## Results

### Demographics

In total, 16 patients (11F, 5M) were recruited and received either real (n = 8) or sham (n = 8) treatment. Of these, 15 (10F, 5M) were included in the analysis. One participant (pp01, F, sham) was excluded after analysis because she was diagnosed with sensory processing sensitivity (SPS). Mean age of our final sample (n = 15) was 44.8y ± 5.8y, with no significant difference between the real (42.5y ± 5.5y) and sham group (47.4y ± 5.3y) (t(12.86) = 1.76, p = 0.103). No participants reported serious adverse events. Only one complained about dizziness at the end of the stimulation.

**Figure 3.**
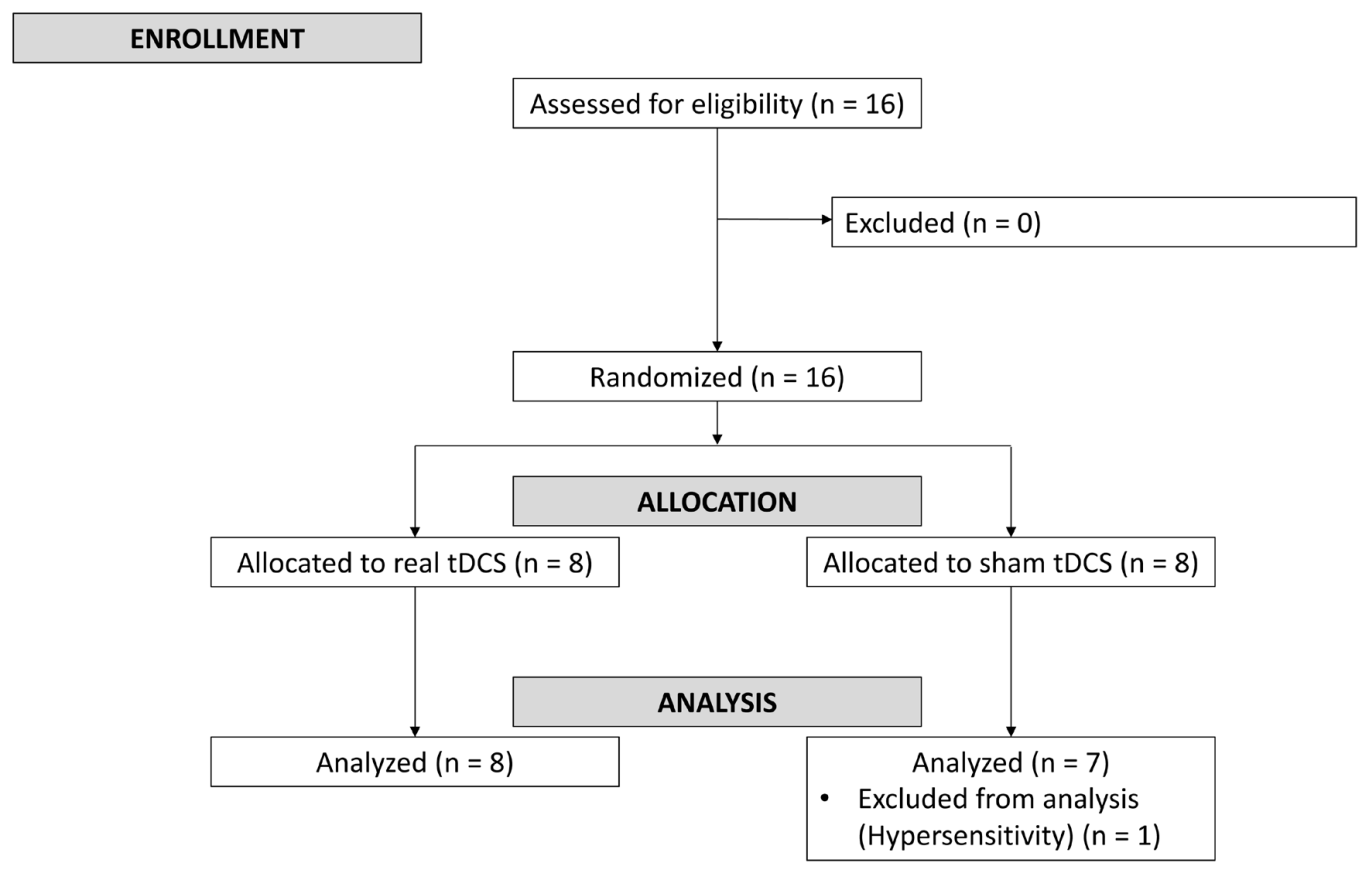
Flow chart of the enrollment procedure (CONSORT 2010 Flow Diagram).

An overview of the demographic characteristics and the initial scores on the MBS, the Dutch version of the BDI, and question A of the Dutch version of the QoL are given in Table 2A (see underlying data ( van Dun, 2020)). Table 2B contains additional demographic characteristics and working-related information. A flow chart is provided in Figure 3.

**Table 2A.** Demographic characteristics of the patients.

Participant	Gender	atDCS	Age	MBS	BDI	QoL
**pp02**	F	Real	40	5.8	35	6
**pp03**	F	Real	52	4.5	21	3
**pp04**	F	Sham	49	4.0	26	9
**pp05**	F	Sham	42	4.2	35	5
**pp06**	F	Real	44	4.4	32	4
**pp07**	F	Real	36	4.0	17	4
**pp08**	F	Real	38	4.1	19	4
**pp09**	F	Real	38	4.5	31	5
**pp10**	M	Sham	38	4.4	24	5
**pp11**	F	Real	48	4.0	24	8
**pp12**	F	Real	44	4.6	20	6
**pp13**	M	Sham	51	6.0	46	1
**pp14**	M	Sham	51	4.0	28	4
**pp15**	M	Sham	52	4.5	19	5
**pp16**	M	Sham	49	4.2	20	6
***# or*** ***Average ± SD***	10F/5M	8 Real/7 Sham	***44.8 ± 5.8***	***4.5 ± 1.0***	***26.5 ± 8.1***	***5.0 ± 1.9***

[i] **Legend**: F = Female; M = Male; MBS = Maslach Burnout Scale; BDI = Beck’s Depression Inventory; tDCS = transcranial Direct Current Stimulation; QoL = McGill Quality of Life Questionnaire; SD = Standard Deviation.

**Table 2B.** Demographic characteristics and working-related information.

Participant	Employment	Education	#working hours at intake	Smokers	Living together/ Children	Antidepressant medication
**pp02**	Fulltime	>12y	70	No	Yes/Yes	OFF
**pp03**	Parttime	12y	0	Yes	Yes/Yes	OFF
**pp04**	Parttime	>12y	14	No	Yes/Yes	OFF
**pp05**	Fulltime	>12y	19	No	Yes/Yes	ON (SNRI)
**pp06**	Parttime	>12y	0	No	Yes/Yes	OFF
**pp07**	Parttime	12y	32	No	Yes/No	OFF
**pp08**	Fulltime	>12y	28	No	Yes/Yes	OFF
**pp09**	Fulltime	>12y	0	No	Yes/Yes	OFF
**pp10**	Fulltime	12y	36	No	No/No	OFF
**pp11**	Parttime	>12y	2	No	Yes/No	OFF
**pp12**	Parttime	>12y	0	No	Yes/Yes	ON (SSRI)
**pp13**	Fulltime	12y	37	No	Yes/Yes	ON (SARI)
**pp14**	fulltime	>12y	45	No	Yes/Yes	OFF
**pp15**	Fulltime	12y	30	No	**Yes/Yes**	OFF
**pp16**	Fulltime	>12y	60	No	**Yes/Yes**	OFF

[i] **Legend**: SARI = Serotonin Antagonist Reuptake Inhibitor; SNRI = Selective Serotonin and Noradrenalin Reuptake Inhibitor; ****SSRI = Selective Serotonin Reuptake Inhibitor; y = years.

### Impact of tDCS on burnout, depressive symptoms, and quality of life

**Figure 4.**
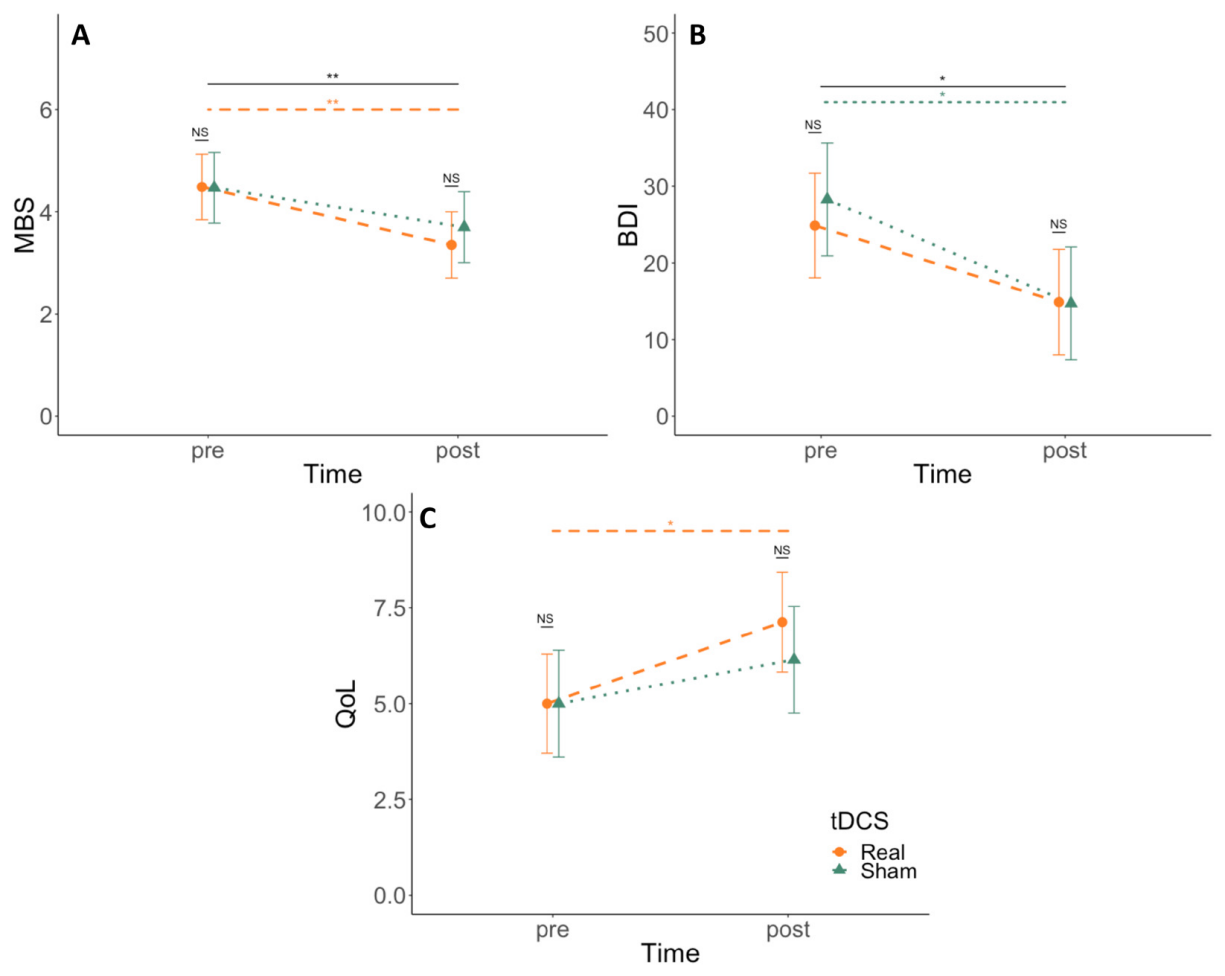
Mean pre- and postscores on **A**. the Maslach Burnout Scale (MBS), **B**. Beck’s Depression Inventory (BDI), and C. McGill Quality of Life (QoL) questionnaire for the sham (dotted, triangles) and real (dashed, circles) group with 95% confidence intervals. Continuous lines indicate main effects, dashed and dotted lines indicate a significant difference between the pre- and postscores of the separate group (real: dashed; sham: dotted) as found by the post-hoc analyses. NS = non-significant; * = p≤0.05; ** = p≤0.01.

The linear mixed model revealed a significant effect of Time for the MBS (F(1, 13) = 15.10, p = 0.002), but no effect of tDCS (F(1, 13) = 0.00, p = 0.971) or an interaction (F(1, 13) = 0.73, p = 0.408) (see Figure 4A). Tukey HSD post-hoc multiple comparisons indicated that only the real tDCS group improved significantly on the MBS (real: t(13) = 3.886, p = 0.009; sham: t(13) = 2.465, p = 0.113).

For the BDI, only an effect of Time was found ((F(1, 13) = 7.93, p = 0.015) (see Figure 4B). Post-hoc analyses revealed that only the sham group improved significantly after the intervention (t(13) = 3.58, p = 0.016) and the real group demonstrated a tendency towards improvement (t(13) = 2.82, p = 0.062).

The linear mixed model revealed no significant effects or interaction for the McGill Quality of Life (QoL) questionnaire. However, post-hoc analysis did reveal a significant improvement for the real group (t(13) = -3.21, p = 0.031) (see Figure 4C).

No significant differences were found at baseline for these three measures (MBS: t(13) = 0.04, p = 1.000; BDI: t(13) = -0.73, p = 0.883; QoL: t(13) = 0.00, p = 1.000). All means, SDs, and p-values of the post-hoc analyses are listed in Table 3. The results of the linear mixed model can be found in Table 7.

**Table 3.** Pre- and postscores and p-values of the burnout and depression assessments and the Quality of Life questionnaire per group.

General	tDCS	pre	p (real vs sham)	post	p (pre vs post)
MBS	real	4.49 ± 0.58	1.000	3.35 ± 0.93	0.009 **
	sham	4.47 ± 0.70		3.70 ± 1.11	0.113
BDI	real	24.88 ± 6.83	0.883	14.88 ± 10.72	0.062
	sham	28.29 ± 9.46		14.71 ± 8.60	0.016 *
QoL	real	5.00 ± 1.60	1.000	7.13 ± 1.13	0.031 *
	sham	5.00 ± 2.38		6.14 ± 1.57	0.406

* : p≤0.05 **: p≤0.01 **Legend**: tDCS = transcranial Direct Current Stimulation; MBS = Maslach Burnout Scale, BDI = Beck’s Depression Inventory; QoL = Quality of Life questionnaire.

### Impact of tDCS on attention and vigilance

**Figure 5.**
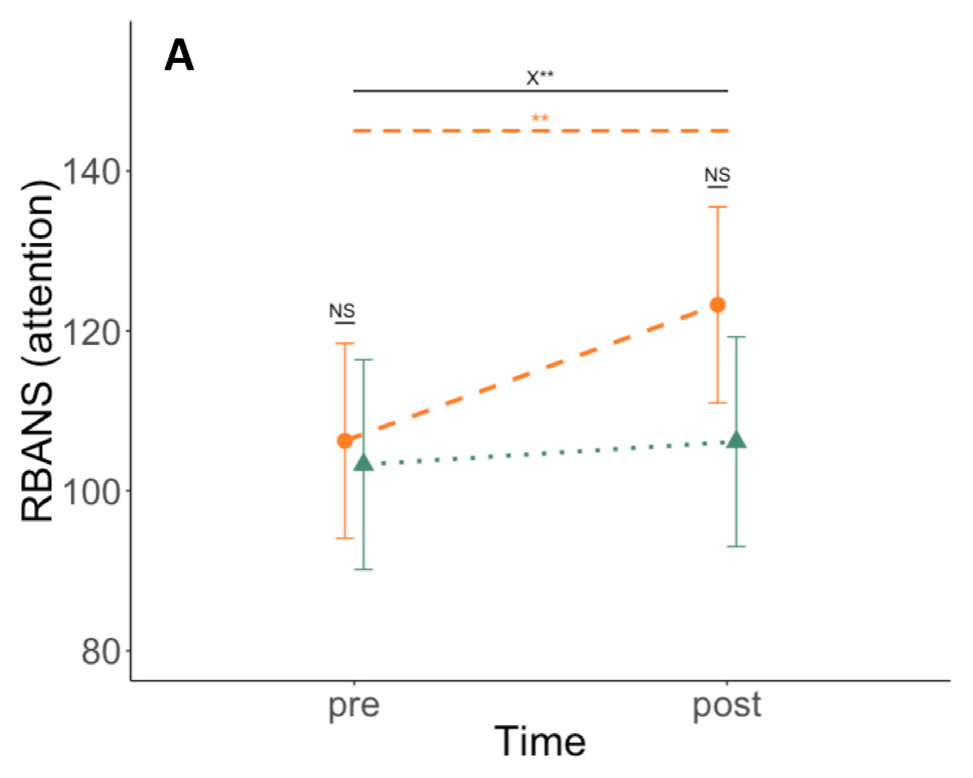
Mean pre- and postscores on the Repeatable Battery for the Assessment of Neuropsychological Status (RBANS) Attention index for the sham (dotted, triangles) and real (dashed, circles) group with 95% confidence intervals. Continuous lines with X indicate interaction effects, dashed and dotted lines indicate a significant difference between the pre- and postscores of the separate group (real: dashed; sham: dotted) as found by the post-hoc analyses. X = interaction effect; NS = non-significant; * = p≤0.05; ** = p≤0.01.

Means, standard deviations, and p-values of the post-hoc analyses are shown in Table 4. The linear mixed model revealed a significant interaction between Time and tDCS for the RBANS Attention Index (F(1,13) = 14.80, p = 0.048), where the real group improved significantly more than the sham group (real: t(13) = -3.85, p = 0.010; sham: t(13) = -0.61, p = 0.929) (see Figure 5A). No significant difference was detected in the baseline scores (t(13) = 0.36, p = 0.984).

**Table 4.** Pre- and postscores and p-values of the attention and vigilance assessments per group.

Attention	tDCS	pre	p (real vs sham)	post	p (pre vs post)
RBANS Attention	real	106.25 ± 15.28	0.984	123.25 ± 10.15	0.010 **
	sham	103.29 ± 16.10		106.14 ± 21.57	0.929
D2 s ^#^	real	80.75 ± 13.58	0.061	72.00 ± 23.27	0.660
	sham	54.43 ± 17.41		84.14 ± 16.09	0.013 *

* : p≤0.05 **: p≤0.01 **Legend**: tDCS = transcranial Direct Current Stimulation; RBANS = Repeatable Battery for the Assessment of Neuropsychological Status; ^#^ = assumptions of the linear mixed model are violated.

A significant interaction effect was also found for vigilance (F(1,13) = 12.15, p = 0.004), as measured by the s-score of the D2 test, with the sham group improving significantly. However, the assumptions of homoscedasticity and normality of the residuals of the model were doubtful, but did not improve using the Box-Cox transformation, which makes it difficult to interpret the results. In addition, the real and sham tDCS group tended to differ significantly at baseline (t(13) = 2.82, p = 0.061), with the sham group performing worse than the real tDCS group.

### Impact of tDCS on the central executive

All means, standard deviations, and p-values of the post-hoc analyses are shown in Table 5.

**Table 5.** Pre- and postscores and p-values of the executive function assessments per group.

Central executive	tDCS	pre	p (real vs sham)	post	p (pre vs post)
**Inhibition & Shifting**					
Stroop Card III	real	47.94 ± 33.90	0.327	64.63 ± 26.67	0.118
	sham	73.57 ± 25.45		77.43 ± 23.44	0.951
WCST	real	2.25 ± 1.49	0.323	3.00 ± 1.20	0.042 *
	sham	3.29 ± 0.76		3.43 ± 0.79	0.948
TMT B ^#^	real	72.25 ± 30.84	0.947	80.63 ± 15.26	0.546
	sham	65.86 ± 25.12		76.14 ± 15.73	0.433
**Updating & Control**					
TMT A	real	52.88 ± 39.08	0.925	70.88 ± 22.77	0.066
	sham	61.86 ± 24.95		75.71 ± 20.61	0.238
Stroop Card I	real	41.00 ± 35.29	0.689	67.75 ± 26.89	0.105
	sham	59.43 ± 29.71		62.14 ± 35.62	0.995
Stroop Card II ^#^	real	49.38 ± 37.65	0.585	55.13 ± 30.77	0.935
	sham	71.29 ± 31.42		69.86 ± 30.43	0.999
D2 G _z_	real	39.81 ± 31.52	0.771	63.64 ± 23.82	0.074
	sham	54.00 ± 30.36		63.57 ± 27.42	0.741
D2 G _z_ – F	real	45.26 ± 33.96	0.596	72.33 ± 20.48	0.059
	sham	62.86 ± 27.30		71.86 ± 22.87	0.812
D2 F%	real	83.13 ± 13.70	0.952	75.00 ± 24.20	0.699
	sham	87.29 ± 9.25		93.29 ± 5.65	0.872

* : p≤0.05 ** : p≤0.01 **Legend**: tDCS = transcranial Direct Current Stimulation; TMT = Trail Making Test; WCST = Wisconsin Card Sorting Test; ^#^ = assumptions of the linear mixed model are violated.

**Figure 6.**
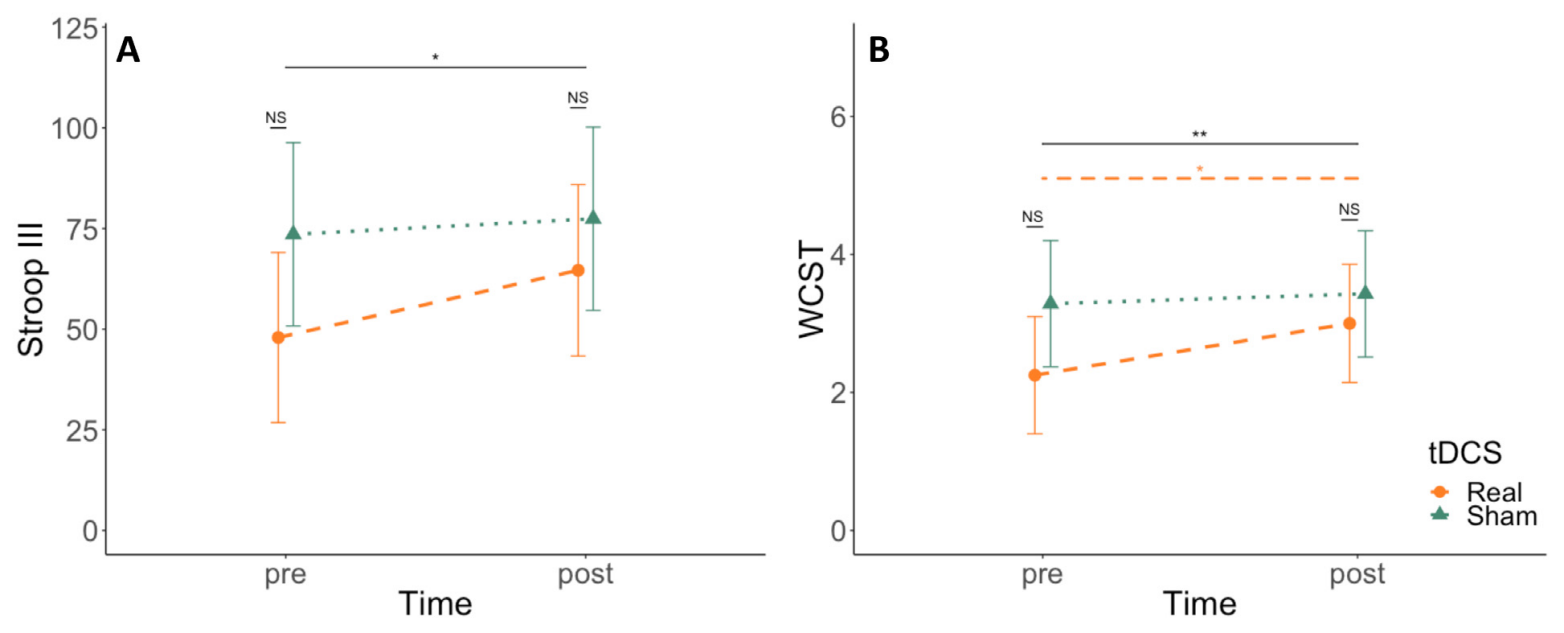
Mean pre- and postscores for inhibition and shifting on **A**. the Stroop Color-Word test Card III, and **B**. the Wisconsin Card Sorting Test (WCST), for the sham (dotted, triangles) and real (dashed, circles) group with 95% confidence intervals. Continuous lines indicate main effects, dashed and dotted lines indicate a significant difference between the pre- and postscores of the separate group (real: dashed; sham: dotted) as found by the post-hoc analyses. NS = non-significant; * = p≤0.05; ** = p≤0.01.

The linear mixed model demonstrated a significant effect of Time for inhibition and shifting on the Stroop Color-Word test (card III) (F(1,13) = 5.96, p = 0.030) ( Figure 6A) and the WCST (F(1,13) = 9.20, p = 0.010) ( Figure 6B). Post-hoc comparisons only revealed a significant improvement in the real tDCS group on the WCST (real: t(13) = -3.03, p = 0.042; sham: t(13) = -0.54, p = 0.948). For the Stroop (card III) no significant improvements were found for either group post-hoc (real: t(13) = -2.44, p = 0.118; sham: t(13) = -0.53, p = 0.951). For the TMT B, the assumption of homoscedasticity of the residuals was violated and did not improve using the Box-Cox transformation, making interpretation of the model difficult. No significant effects or interaction were found with the non-transformed data.

No significant differences were found in the baseline measures (Stroop card III: t(13) = -1.78, p = 0.327; WCST: t(13) = -1.79, p = 0.323; TMT B: t(13) = 0.54, p = 0.947).

**Figure 7.**
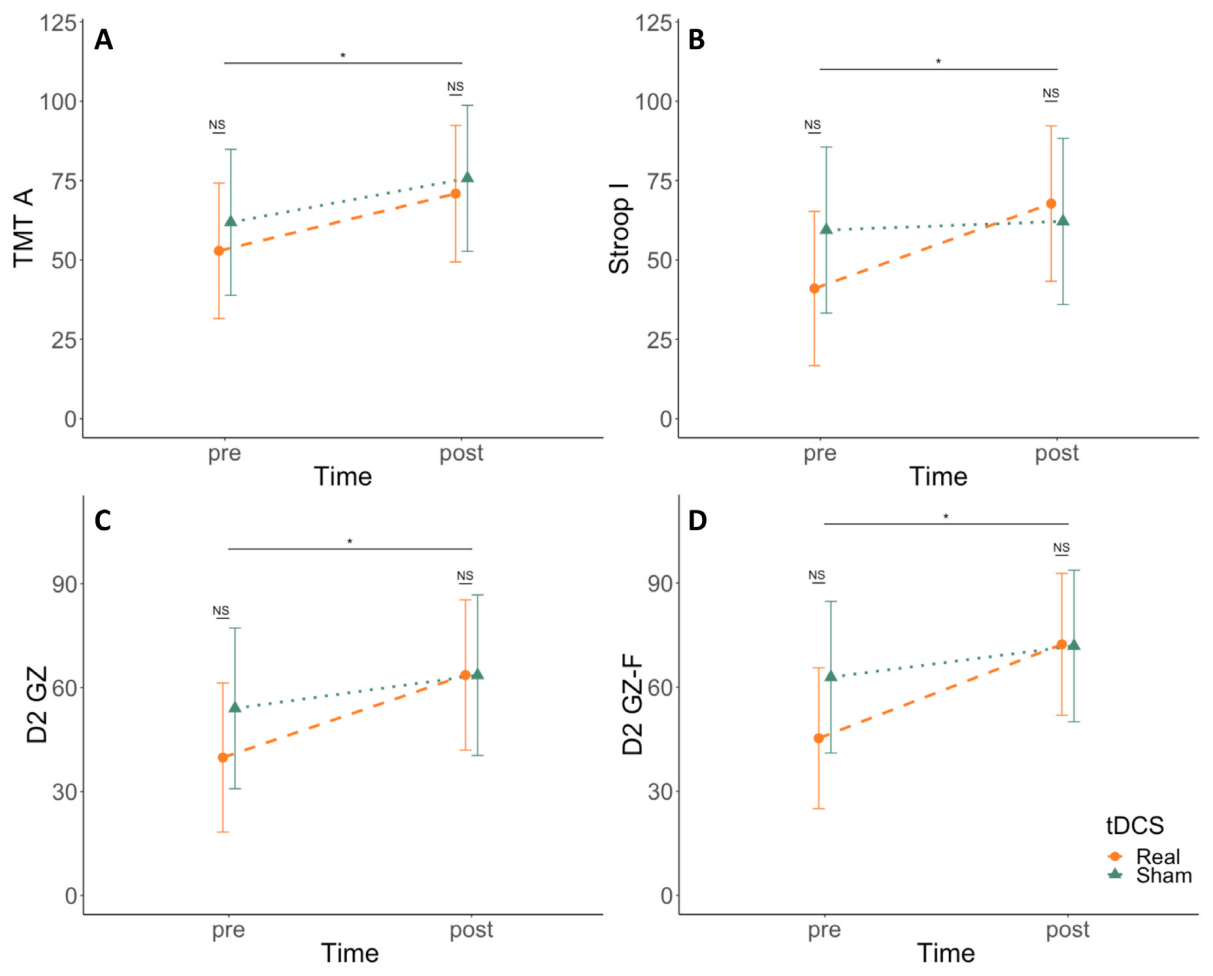
Mean pre- and postscores for updating and control on the **A**. Trail Making Test (TMT) part **A**, **B**. Stroop-Color Word test Card I, C. D2 G _z_ score, and D. D2 G _z _– F score for the sham (dotted, triangles) and real (dashed, circles) group with 95% confidence intervals. Continuous lines indicate main effects, dashed and dotted lines indicate a significant difference between the pre- and postscores of the separate group (real: dashed; sham: dotted) as found by the post-hoc analyses. NS = non-significant; * = p≤0.05; ** = p≤0.01.

For updating and control, a significant effect of Time was found for the TMT A (F(1,13) = 7.69, p = 0.016) ( Figure 7A), the Stroop Color-Word test (card I) (F(1,13) = 6.30, p = 0.026) ( Figure 7B) and the D2 (G _z_: F(1,13) = 7.38, p = 0.018; and G _z_ – F: F(1,13) = 8.10, p = 0.014) ( Figure 7C and 7D). The post-hoc tests only revealed trends towards improvement in the real group for the TMT A (real: t(13) = -2.77, p = 0.067; sham: t(13) = -2.00, p = 0.238), the Stroop Color-Word test (card I) (real: t(13) = -2.51, p = 0.105; sham: t(13) = -0.24, p = 0.995), D2 G _z_ (real: t(13) = -2.72, p = 0.074; sham: t(13) = -1.02, p = 0.741), and D2 G _z_ – F (real: t(13) = -2.85, p = 0.059; sham: t(13) = -0.89, p = 0.812). No significant effects or interaction was found for the Stroop Color-Word test (card II) or F% of the D2 test. However, the assumption of homoscedasticity of the residuals was violated in the Stroop Color-Word test (card II), which might have resulted in unreliable p-values.

No significant differences were found in the baseline measures (TMT A: t(13) = 0.62, p = 0.925; Stroop card I: t(13) = -1.11, p = 0.689; Stroop card II: ; D2 G _z_: t(13) = -0.97, p = 0.771; D2 G _z_ – F: t(13) = -1.27, p = 0.596; D2 F%: t(13) = -0.52, p = 0.952).

### Impact of tDCS on other working memory components

All mean scores, standard deviations, and p-values of the post-hoc analyses are listed in Table 6.

**Table 6.** Pre- and postscores and p-values of the post-hoc analyses of the other working memory components per group.

Working memory	tDCS	pre (Standard Scores)	p (real vs sham)	post (Standard Scores)	p (pre vs post)
**Phonological loop**					
RBANS Language	real	110.00 ± 6.82	0.352	116.00 ± 6.59	0.180
	sham	102.29 ± 9.43		104.57 ± 11.43	0.863
BNT	real	0.34 ± 0.55	0.995	1.30 ± 0.67	0.015 *
	sham	0.27 ± 0.79		1.08 ± 0.30	0.060
Semantic fluency	real	82.50 ± 7.45	0.921	89.50 ± 11.01	0.416
	sham	77.86 ± 18.21		88.43 ± 18.58	0.162
**Visuospatial sketchpad**					
Raven ^#^	real	123.00 ± 4.21	0.999	124.50 ± 3.96	0.915
	sham	123.43 ± 6.48		125.00 ± 6.14	0.920
RBANS Visuospatial Memory	real	115.88 ± 8.31	0.355	121.25 ± 6.11	0.452
	sham	107.43 ± 12.78		110.86 ± 10.22	0.800
**Encoding**					
RBANS Immediate Memory	real	109.00 ± 8.98	0.986	124.25 ± 14.34	0.020 *
	sham	106.29 ± 17.31		113.00 ± 20.15	0.508
**Retrieval**					
RBANS Recent Memory	real	103.88 ± 11.19	0.943	113.75 ± 9.51	0.051
	sham	106.71 ± 8.52		116.00 ± 9.87	0.094

* : p≤0.05 ** : p≤0.01 **Legend**: tDCS = transcranial Direct Current Stimulation; RBANS = Repeatable Battery for the Assessment of Neuropsychological Status; BNT = Boston Naming Test; ^#^ = assumptions of the linear mixed model are violated.

**Figure 8.**
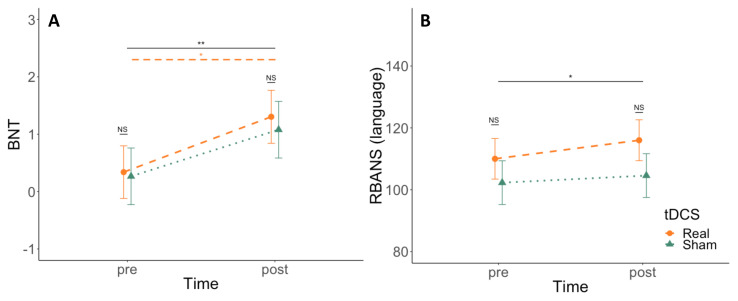
Mean pre- and postscores for the phonological loop on the **A**. Boston Naming Test (BNT), and **B**. RBANS Language index for the sham (dotted, triangles) and real (dashed, circles) group with 95% confidence intervals. Continuous lines indicate main effects, dashed and dotted lines indicate a significant difference between the pre- and postscores of the separate group (real: dashed; sham: dotted) as found by the post-hoc analyses. NS = non-significant; * = p≤0.05; ** = p≤0.01.

For the phonological loop, the linear mixed model revealed a main effect of Time for the BNT (F(1,13) = 12.92, p = 0.003) ( Figure 8A) and the Language index of the RBANS (F(1,13) = 4.76, p = 0.048) ( Figure 8B). Tukey HSD post-hoc comparisons revealed that only the real tDCS group improved significantly on the BNT (t(13) = -3.60, p = 0.015), a trend towards improvement was observed in the sham group (t(13) = -2.83, p = 0.060). No significant improvements were found for the groups separately for the RBANS Language Index (real: t(13) = -2.18, p = 0.180; sham: t(13) = 0.78, p = 0.863). No significant effects or interactions were seen for semantic fluency. Baseline scores did not differ significantly for these measures (BNT: t(13) = 0.23, p = 0.995; RBANS Language Index: t(13) = 1.72, p = 0.352; Semantic fluency: t(13) = 0.63, p = 0.921).

No significant effects or interaction were observed for the visuospatial sketchpad (RBANS Visuospatial index and Raven). However, the assumptions for the linear mixed model of the Raven were violated, which could have affected the p-values. The Box-Cox transformation did not improve the data. Baseline scores did not differ significantly (RBANS Visuospatial Index: t(13) = 1.72, p = 0.452; Raven: t(13) = -0.16, p = 0.999).

**Figure 9.**
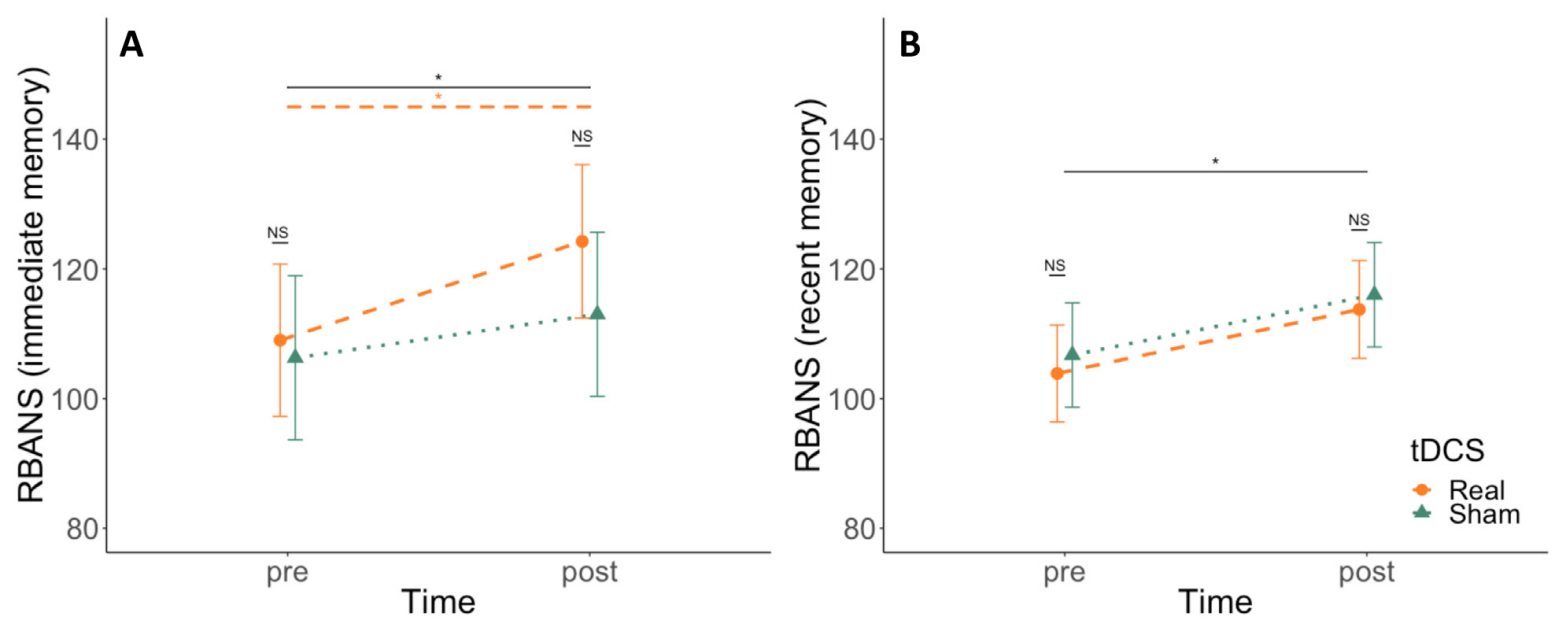
Mean pre- and postscores for encoding on the **A**. Repeatable Battery for the Assessment of Neuropsychological Status (RBANS) Immediate Memory, and retrieval on the **B**. RBANS Recent Memory for the sham (dotted, triangles) and real (dashed, circles) group with 95% confidence intervals. Continuous lines indicate main effects, dashed and dotted lines indicate a significant difference between the pre- and postscores of the separate group (real: dashed; sham: dotted) as found by the post-hoc analyses. NS = non-significant; * = p≤0.05; ** = p≤0.01.

A main effect of Time was observed for encoding, evaluated by the Immediate Memory index of the RBANS (F(1,13) = 11.93, p = 0.004) ( Figure 9A). Post hoc analysis showed that this was mainly driven by a significant improvement of the real tDCS group (real: t(13) = -3.45, p = 0.020; sham: t(13) = -1.42, p = 0.508). Retrieval (RBANS Recent Memory index) also showed a significant effect of Time (F(1,13) = 8.58, p = 0.012) ( Figure 9B). For retrieval, both groups trended towards significance (real: t(13) = -2.93, p = 0.051; sham: t(13) = -2.58, p = 0.094). No differences in baseline scores were observed for Immediate or Recent Memory (RBANS Immediate Memory Index: t(13) = 0.34, p = 0.986; RBANS Recent Memory Index: t(13) = -0.56, p = 0.943).

**Table 7.** F- and p-values for the linear mixed models.

Test	Effect	F(1, 13)	p
**GENERAL**			
MBS	Time tDCS Time x tDCS	15.10 0.00 0.73	0.002 ** 0.971 0.408
BDI	Time tDCS Time x tDCS	7.93 0.53 0.47	0.015 * 0.478 0.504
QoL	Time tDCS Time x tDCS	2.60 0.00 1.02	0.131 1.000 0.330
**ATTENTION**			
RBANS Attention	Time tDCS Time x tDCS	14.80 0.13 4.78	0.002 ** 0.727 0.048 *
D2 s ^#^	Time tDCS Time x tDCS	1.35 7.96 12.15	0.267 0.014 * 0.004 *
**CENTRAL EXECUTIVE** *Inhibition & Shifting*			
Stroop Card III	Time tDCS Time x tDCS	5.96 3.16 1.64	0.030 * 0.099 0.222
WCST	Time tDCS Time x tDCS	9.20 3.19 2.81	0.010 ** 0.097 0.117
TMT B ^#^	Time tDCS Time x tDCS	1.84 0.29 0.04	0.198 0.598 0.836
*Updating & Control*			
TMT A	Time tDCS Time x tDCS	7.69 0.38 0.19	0.016 * 0.548 0.670
Stroop Card I	Time tDCS Time x tDCS	6.30 1.24 2.37	0.026 * 0.287 0.147
Stroop Card II ^#^	Time tDCS Time x tDCS	0.34 1.66 0.25	0.568 0.220 0.626
D2 Gz	Time tDCS Time x tDCS	7.38 0.93 1.23	0.018 * 0.352 0.287
D2 Gz – F	Time tDCS Time x tDCS	8.10 1.61 1.68	0.014 * 0.226 0.217
D2 F%	Time tDCS Time x tDCS	1.20 0.28 1.69	0.294 0.609 0.216
**WORKING MEMORY** *Phonological loop*			
RBANS Language	Time tDCS Time x tDCS	4.76 2.97 0.85	0.048 * 0.109 0.373
BNT	Time tDCS Time x tDCS	12.92 0.05 0.15	0.003 ** 0.819 0.705
Semantic fluency	Time tDCS Time x tDCS	2.53 0.40 0.31	0.136 0.541 0.589
*Visuospatial sketchpad*			
Raven ^#^	Time tDCS Time x tDCS	0.42 0.03 0.00	0.530 0.877 0.984
RBANS Visuospatial Memory	Time tDCS Time x tDCS	2.32 2.95 0.14	0.151 0.110 0.712
**ENCODING**			
RBANS Immediate Memory	Time tDCS Time x tDCS	11.93 0.11 1.75	0.004 ** 0.740 0.209
**RETRIEVAL**			
RBANS Recent Memory	Time tDCS Time x tDCS	8.58 0.31 0.01	0.012 * 0.588 0.907

*: p≤0.05 **: p≤0.01 **Legend**: tDCS = transcranial Direct Current Stimulation; MBS = Maslach Burnout Scale; BDI = Beck’s Depression Inventory; RBANS = Repeatable Battery for the Assessment of Neuropsychological Status; TMT = Trail Making Test; WCST = Wisconsin Card Sorting Test; BNT = Boston Naming Test; ^#^ = assumptions of the linear mixed model are violated.

## Discussion

This randomized blinded sham-controlled study investigated the impact of daily atDCS sessions (2mA, 20min) over the left DLPFC (AF3) with the reference over the contralateral orbit (F8) on attention and the central executive, as well as other components of the working memory in patients with burnout. This electrode montage has been shown to be effective in patients with depression, showing not only an antidepressant effect but also a positive effect on mood, attention skills, and working memory ( Loo *et al*., 2012). We included 15 patients (7 sham, 8 tDCS) in a 3-week protocol and investigated their cognitive and attention skills, as well as their burnout severity, depression, and overall quality of life before and after treatment. Both groups improved on all these measures, which can be expected due to the behavioral therapy both groups received, but the improvement of burnout and overall quality of life was only significant after real tDCS. Surprisingly, however, only the sham group significantly improved on the depression scale. This might be due to the fact that depression scores were moderate, while in the study of Loo *et al*. only patients with a DSM IV major depression episode were included ( Loo *et al*., 2012). Moreover, in the study of Loo *et al*. depression was rated by an experienced psychiatrist/psychologist using the Montgomery Asberg Depression Rating Scale (MADRS; Montgomery & Asberg, 1979), while in our study a self-assessment scale (BDI) was used ( Loo *et al*., 2012).

For the main variable of interest ( **Attention** index of the RBANS), a significant interaction between tDCS and Time was found, showing that real anodal tDCS over the left DLPFC can have an added value to conventional therapy in the rehabilitation of attention in burnout patients.

It is known that burnout primarily impairs functions of the **central executive**, whereas brain areas that regulate other components of the working memory are affected to a lesser degree. The central executive -mainly located in the prefrontal brain regions- could be the component that is most vulnerable to chronic stress because its higher order attention control functions are more demanding and complex than those performed by the other subcomponents ( Deligkaris *et al*., 2014). Several studies investigating the impact of burnout on cognitive functions have confirmed that the most pronounced differences between patients and controls were seen on tests that are highly dependent upon the executive functions, e.g. prospective memory, processing speed, complex working memory, sustained attention, and letter fluency ( Eskildsen *et al.*, 2015; Jonsdottir *et al*., 2013; Öhman *et al.*, 2007; Orena *et al.*, 2013)(35–38)( Eskildsen *et al.*, 2015; Jonsdottir *et al*., 2013; Öhman *et al.*, 2007; Orena *et al.*, 2013). This study showed that three weeks of therapy, combined with real or sham stimulation, significantly improved several components of the central executive. However, analyses revealed that improvement was driven by a significant improvement after real tDCS for inhibition and shifting (WCST), and was also primarily seen after real tDCS for updating and control (TMT A, D2 G _z_, D2 G _z_ – F). These results are in line with the study of Miler *et al*. who found that atDCS over the left DLPFC significantly improves the executive control of attention ( Miler *et al*., 2018). As in the study of Miler *et al*., no effect was seen on the percentage of mistakes, but the processing speed did improve more on the D2 test of attention after real tDCS than after sham tDCS ( Miler *et al*., 2018). However, the improvement on inhibition and shifting after tDCS was somewhat unexpected, since this is primarily associated with the right DLPFC and the right inferior frontal gyrus ( Lie *et al*., 2006; Hampshire *et al*., 2010). Since this effect was only observed for the WCST, this might be related to this specific task. Indeed, studies have shown that, although the right DLPFC seems to be the most prominent in handling complex/manipulative working memory operations in the WCST ( Lie *et al*., 2006), the left DLPFC is also involved during this task ( Lie *et al*., 2006; Nagahama *et al*., 2005). 

atDCS also seemed to have a positive impact on other components of the working memory. The phonological loop might also be positively influenced by real tDCS as shown by a significant improvement of the real tDCS group on the BNT, although the sham group also trended towards a significant improvement. No effect was seen on the visuospatial sketchpad, which might not be surprising because this is believed to be situated primarily in the right prefrontal cortex ( Suchan, 2008), but encoding clearly improved more after real atDCS than after sham tDCS (RBANS Immediate Memory Index). Transcranial magnetic stimulation (TMS) studies have shown a prominent role for the left DLPFC during encoding, observing shorter reaction times using a paired-pulse paradigm over this area ( Gagnon *et al*., 2011). Though de Lara *et al*. (2017) did not find any effect of anodal tDCS over the left DLPFC on encoding, this might be due to the lesser intensity (1mA vs 2mA) they used in their study. 

These data provide preliminary evidence for the value of tDCS over the left DLPFC in rehabilitating attention deficits, and possibly also central executive and encoding deficits, in burnout patients.

## Limitations and conclusion

Our study has several important limitations. First, our group of patients was relatively small. This is an important limitation given the positive trends of the effect of real tDCS on several outcome measures. Studies with more power will have to show whether these trends failed to reach significance due to a lack of power. In addition, some variables of interest (D2 s-score, TMT B, Stroop Card II, Raven) could not be interpreted correctly with the linear mixed model analysis because of a violation of assumptions. More data points could help to resolve this issue. Setting up multi-site cooperations to recruit participants and maintaining close relationships with primary care providers making them aware of the safety of tDCS when applied in the correct manner, could also help to convince patients to participate in tDCS studies. Larger groups to validate the efficacy of tDCS are crucial to investigate the clinical usability of this therapeutic aid.

Second, patients were randomized over both groups, which led to an overrepresentation of men in the sham group. At the moment, it is not clear whether gender can have a significant impact on the effect of tDCS ( Antal *et al*., 2017), or whether there are gender-related differences in the symptoms of burnout ( Purvanova & Muros, 2010), but this imbalance of gender between both groups might have affected the results.

Third, our group of patients was very heterogeneous. For example, the moment of participation in the study was variable during the burnout process. Some participants were still at work, others were not yet able to start working, others were already re-integrated in their jobs. Due to the sample size, it was not possible to investigate the effects of different factors, such as living circumstances, age, gender, education, etc. on the progress of burnout. In addition, three of the patients were taking antidepressant medication during the study, of whom one received real stimulation. It has been shown that this type of medication (selective serotonin reuptake inhibitors or SSRIs) might enhance the LTP-like plasticity induced by anodal tDCS ( Kuo *et al*., 2016). Future studies should focus on these parameters to elucidate the influence of these factors on burnout recovery and on tDCS outcome. In addition, it is recommended to test for the efficiency of blinding the type of stimulation by asking the participants afterwards whether they think they were actively stimulated or not. Gathering information about the amount of discomfort could also be of importance for future studies using tDCS.

Fourth, the placement of the electrodes might not have been optimal to target attention deficits. Our study was based on the outcome of Loo *et al*. (2012) who aimed to investigate the anti-depressant effect in patients with depression, but found an improvement of attention and working memory instead ( Loo *et al*., 2012). By copying this electrode placement, we hoped to replicate these results in patients with burnout. However, by placing the cathode on F8, we might have unwantedly inhibited the right inferior frontal gyrus, which has been linked to inhibition and attentional control ( Hampshire *et al*., 2010). Although cathodal tDCS over the right inferior frontal gyrus did not appear to have a significant effect on response stopping or reaction times in a stop-signal task ( Stramaccia *et al*., 2015), another choice for the cathodal reference electrode might be warranted. In addition, AF3 targets primarily the more frontal site of the left DLPFC, while a more common placement to target DLPFC in attention studies is F3 ( Coffman *et al*., 2014).

Lastly, research has shown that the effect of tDCS on working memory might be dependent on, amongst others, the initial dopaminergic level that can impact the excitation/inhibition balance (i.e. homeostasis between relative contributions of excitatory and inhibitory synaptic inputs) ( Polizzotti *et al*., 2020). More insight into the exact working mechanisms underlying the cognitive and attention deficits in burnout patients might be beneficial for future research.

Despite these shortcomings, these data provide preliminary evidence for the value of atDCS over the left DLPFC in rehabilitating attention deficits in burnout. tDCS might prove to be a useful, affordable, and easy-to-use addition to conventional therapy to speed up reintegration of burnout patients.

## Consent

Written informed consent for publication of the patients’ details was obtained from the patients.

## Data availability

### Underlying data

Harvard Dataverse: Transcranial direct current stimulation and attention skills in burnout patients: a randomized blinded sham-controlled pilot study. https://doi.org/10.7910/DVN/4VG2XS ( van Dun, 2020)

This project contains the following underlying data:

- Data_burnout.txt (Data used for statistical analyses)
- Raw Data Burnout.tab (Raw data for the Burnout study (Raw Scores (RS) and Standard Scores (SS)))

### Extended data

Harvard Dataverse: Transcranial direct current stimulation and attention skills in burnout patients: a randomized blinded sham-controlled pilot study. https://doi.org/10.7910/DVN/4VG2XS ( van Dun, 2020)

This project contains the following extended data:

- Verbal_fluency.pdf (Means and standard deviations per age, gender, and educational level of 200 Dutch-speaking participants for the verbal (semantic) fluency task)

### Reporting guidelines

CONSORT checklist and flow chart for “Transcranial direct current stimulation and attention skills in burnout patients: a randomized blinded sham-controlled pilot study”. https://doi.org/10.7910/DVN/4VG2XS ( van Dun, 2020)

Data are available under the terms of the Creative Commons Zero “No rights reserved” data waiver (CC0 1.0 Public domain dedication).
