# Supplementary material for: Transcranial direct current stimulation and attention skills in burnout patients: a randomized blinded sham-controlled pilot study
Source: F1000Res. 2020 Jul 31;9:116. Originally published 2020 Feb 14. [Version 2] doi: 10.12688/f1000research.21831.2 (PMC7737710; doi:10.12688/f1000research.21831.2)
Supplement: Peer review history

# Peer review: Reviewer response for version 2

**DOI:** 10.5256/f1000research.28282.r74299

**Published:** 2020-12-14

**Authors:** Hongxing Wang

**Affiliations:** 1 Division of Neuropsychiatry and Psychosomatics, Department of Neurology of Xuanwu Hospital, Capital Medical University, Beijing, China

It is an interesting piece of work; however, I have some concerns:

1. The manuscript does not include the current literature, such as:
  Wang HX, Wang L, Zhang WR, et al. Effect of Transcranial Alternating Current Stimulation for the Treatment of Chronic Insomnia: A Randomized, Double-Blind, Parallel-Group, Placebo-Controlled Clinical Trial. Psychother Psychosom. 2020;89(1):38-47. doi: 10.1159/000504609. Epub 2019 Dec 17. PMID: 31846980. ^1^
2. Sample is the main limitation, therefore, the title is recommended to change into the following:
  "Transcranial direct current stimulation and attention skills in burnout patients: a proof of concept study using a randomly blinded design"
3. Limitations section of Discussion should be further summarized and merged for a more concise and clear style, from reader's view.

Is the work clearly and accurately presented and does it cite the current literature?

Partly

If applicable, is the statistical analysis and its interpretation appropriate?

I cannot comment. A qualified statistician is required.

Are all the source data underlying the results available to ensure full reproducibility?

Yes

Is the study design appropriate and is the work technically sound?

Yes

Are the conclusions drawn adequately supported by the results?

Yes

Are sufficient details of methods and analysis provided to allow replication by others?

Partly

Reviewer Expertise:

psychiatry, neuroscience, neuropsychoneurology. psychosomatics, neuromodulation, transcranial alterative current stimulation in different patients affected with brain disorders and psychitrical disorders.

I confirm that I have read this submission and believe that I have an appropriate level of expertise to confirm that it is of an acceptable scientific standard.

## References

[1] : Effect of Transcranial Alternating Current Stimulation for the Treatment of Chronic Insomnia: A Randomized, Double-Blind, Parallel-Group, Placebo-Controlled Clinical Trial. *Psychother Psychosom*.2020;89(1) : 10.1159/000504609 38-47 10.1159/000504609 31846980

# Peer review: Reviewer response for version 2

**DOI:** 10.5256/f1000research.28282.r68460

**Published:** 2020-08-17

**Authors:** Choi Deblieck

**Affiliations:** 1 UPC KU Leuven, Gasthuisberg, Belgium

I read through the revised version of the paper, and conclude that the authors have addressed my comments in a satisfactory way.

Is the work clearly and accurately presented and does it cite the current literature?

Partly

If applicable, is the statistical analysis and its interpretation appropriate?

Partly

Are all the source data underlying the results available to ensure full reproducibility?

Partly

Is the study design appropriate and is the work technically sound?

Partly

Are the conclusions drawn adequately supported by the results?

Partly

Are sufficient details of methods and analysis provided to allow replication by others?

Partly

Reviewer Expertise:

non-invasive neuromodulation (TMS/tDCS), fMRI

I confirm that I have read this submission and believe that I have an appropriate level of expertise to confirm that it is of an acceptable scientific standard.

# Peer review: Reviewer response for version 1

**DOI:** 10.5256/f1000research.24067.r63825

**Published:** 2020-06-03

**Authors:** Choi Deblieck

**Affiliations:** 1 UPC KU Leuven, Gasthuisberg, Belgium

"Transcranial direct current stimulation and attention skills in burnout patients: a randomized blinded sham-controlled pilot study" is addressing an important research and clinical question regarding executive memory, attention, and clinical symptoms in burnout. Data from 15 patients, randomized to 3 weeks of active anodal tDCS over left DLPFC or sham combined with standard behavioral therapy revealed that anodal tDCS significantly improved attention in patients with burnout.  

**Major Comments:**

 Major problems with neuromodulation studies are the small sample sizes and heterogeneous patient populations. The authors already mentioned those pitfalls as their biggest shortcomings. Nonetheless, it remains a major point of criticism for me. In order to get significance in TMS (and tDCS studies), Sack *et al.*, (2009) ^1^ demonstrated that power analyses showed 5 participants are sufficient to reveal a significant behavioral effect on cognition in TMS studies using fMRI-guided neuronavigation. However, the number of necessary participants increases to n = 9 in studies using MRI-guided neuronavigation, to n = 13 when using group Talairach coordinates, and to n = 47 when using EEG positions, like F3 or AF3.

 As for the limitation of using heterogeneous patient groups, the demographic characteristics revealed are limited to age, gender, and burnout severity expressed by MBS. Reporting other relevant characteristics, such as duration, employment, ON/OFF medication, etiology, level of education, hospitalization, etc,… would give us a better understanding of how representative the groups were. Also, some of these variables could be explored for significance or even predictivity in future studies.

 Also, since a trend towards inhibition and shifting was reported, and the right inferior frontal gyrus has been associated with inhibition, the choice of placing the cathode on F8 was not optimal. rIFG is only about 2 to 2.5 cm posterior to F8. Since the reference sponge used had a size of 5x7, rIFG could have been affected by the current.

 Only continuous outcome measures were reported. Since it better reflects better clinical practice, I would add a categorical outcome measure, i.e., percentage of responders/remitters.

 A TCT device was used. The strap it comes with is made of a neoprene strap which could absorb the saline solution potentially increasing the surface area of the target area. Also, is this device EC approved?

 It was unclear whether all the patients in both conditions had been undergoing psychotherapy prior to the add-on tDCS therapy. If psychotherapy was new to all, it is a major confound. It could also be the reason why both groups improved burnout and depression-wise.

 In the tDCS protocol section, Fp3 is mentioned as the target site, “left DLPFC”. Since the 10-20 system of electrode placement is used, defining the target site as AF3 is probably better than Fp3. Also, the vast majority of TMS/tDCS studies targeting left DLPFC to stimulate F3. Why was Fp3/AF3 selected? DLPFC is indeed a large area. But an elaboration on the reason why a more anterior part of DLPFC was chosen should be explained.

**Minor Comments:**

 In the background section of the abstract/methods, only one objective (attention and executive control) is mainly elaborated upon. I would also add the clinical objective, i.e., burnout, depression amelioration. Only QoL was briefly mention.

 The tDCS section of the introduction is not well structured. It’s better to subdivide the use of tDCS into neurological and psychiatric conditions. Now a distinction is made between “motor, cognitive, affective disorders” and “Alzheimer’s and MDD”.

 Two papers on depression are mentioned: one submitting patients to 1 session and the other to 15 sessions. I would talk about the importance of the number of sessions as a potential variable that could increase tDCS effect (Brunoni) ^2^ to explain why you submitted the patients to 15 sessions. Also, there are maybe one or two articles on stress in professionals, indirectly referring to burnout with no tDCS effect on burnout. One of the contributions of this article is that it may be the first looking at burnout. I would highlight this.

 I would also go more into details of other potential brain regions that could have been targeted, especially for those tasks that did not show a tDCS effect. Only DLPFC is mentioned. E.g. as mentioned above, inhibition has not been linked to DLPFC. Or why you included those tasks while targeting DLPFC. It could be the reason why no significance was observed in inhibition.

 A paragraph on neurotransmitters could also be added. Specifically, alterations in dopamine and noradrenaline levels in DLPFC have been associated with impaired working memory performance. TMS and tDCS over DLPFC have also been shown to release dopamine in various brain regions, including the right ventral striatum. Further, tDCS to DLPFC has shown to improve participants’ memory accuracy, an effect that has been correlated significantly with dopamine release.

 I would also mention briefly why it took 3 years to recruit 15 patients. It could be helpful for future studies recruiting patients with burnout.

 Were the patients asked at the end of the treatment whether they knew they had received sham or real? (chi-squared test to test the integrity of masking).

 Were patients asked to evaluate their level of discomfort? The level of discomfort/pain has been associated with a TMS/tDCS effect.

Is the work clearly and accurately presented and does it cite the current literature?

Partly

If applicable, is the statistical analysis and its interpretation appropriate?

Partly

Are all the source data underlying the results available to ensure full reproducibility?

Partly

Is the study design appropriate and is the work technically sound?

Partly

Are the conclusions drawn adequately supported by the results?

Partly

Are sufficient details of methods and analysis provided to allow replication by others?

Partly

Reviewer Expertise:

non-invasive neuromodulation (TMS/tDCS), fMRI

I confirm that I have read this submission and believe that I have an appropriate level of expertise to confirm that it is of an acceptable scientific standard, however I have significant reservations, as outlined above.

## References

[1] : Optimizing functional accuracy of TMS in cognitive studies: a comparison of methods. *J Cogn Neurosci*.2009;21(2) : 10.1162/jocn.2009.21126 207-21 10.1162/jocn.2009.21126 18823235

[2] : Clinical research with transcranial direct current stimulation (tDCS): challenges and future directions. *Brain Stimul*.2012;5(3) : 10.1016/j.brs.2011.03.002 175-195 10.1016/j.brs.2011.03.002 22037126PMC3270156

# Response

**Published:** 2020-07-22

**Authors:** Kim van Dun

**Affiliations:** UHasselt, Belgium

"Transcranial direct current stimulation and attention skills in burnout patients: a randomized blinded sham-controlled pilot study" is addressing an important research and clinical question regarding executive memory, attention, and clinical symptoms in burnout. Data from 15 patients, randomized to 3 weeks of active anodal tDCS over left DLPFC or sham combined with standard behavioral therapy revealed that anodal tDCS significantly improved attention in patients with burnout.  

**Major Comments:**

Major problems with neuromodulation studies are the small sample sizes and heterogeneous patient populations. The authors already mentioned those pitfalls as their biggest shortcomings. Nonetheless, it remains a major point of criticism for me. In order to get significance in TMS (and tDCS studies), Sack *et al.*, (2009) ^1^ demonstrated that power analyses showed 5 participants are sufficient to reveal a significant behavioral effect on cognition in TMS studies using fMRI-guided neuronavigation. However, the number of necessary participants increases to n = 9 in studies using MRI-guided neuronavigation, to n = 13 when using group Talairach coordinates, and to n = 47 when using EEG positions, like F3 or AF3.

***Response: **This is indeed a major shortcoming of our study but unfortunately, as a private practice, we do not have the facilities to set up a big study. Nonetheless, we do think our results are noteworthy and we therefore referred to our study as a “pilot study” hoping to instigate more interest in burnout in the field of non-invasive stimulation. In addition, since tDCS is not as focal as TMS, we do not think the use of EEG positions instead of neuronavigation will impact the power of the study as much as for TMS.*

As for the limitation of using heterogeneous patient groups, the demographic characteristics revealed are limited to age, gender, and burnout severity expressed by MBS. Reporting other relevant characteristics, such as duration, employment, ON/OFF medication, etiology, level of education, hospitalization, etc,… would give us a better understanding of how representative the groups were. Also, some of these variables could be explored for significance or even predictivity in future studies.

***Response: **We did collect other demographic characteristics and have added these to the article to help future studies unravel the significance of these characteristics. Table 2B: Demographic characteristics and working-related information shows Employment (parttime or fulltime), Education level (12y of >12y), #working hours at intake, Smokers (yes or no), Living together / Children (yes or no), and Medication. Since three patients were on antidepressants (SSRI, SNRI, or SARI), of whom one (SSRI) received real stimulation, it has also been added to the limitations section that this might have impacted the outcome of this patient since it has been shown that chronic use of SSRIs can enhance the effect of tDCS.*

*“In addition, three of the patients were taking antidepressant medication during the study, of whom one received real stimulation. It has been shown that this type of medication (selective serotonin reuptake inhibitors or SSRIs) might enhance the LTP-like plasticity induced by anodal tDCS (Kuo et al., 2016).”*

Also, since a trend towards inhibition and shifting was reported, and the right inferior frontal gyrus has been associated with inhibition, the choice of placing the cathode on F8 was not optimal. rIFG is only about 2 to 2.5 cm posterior to F8. Since the reference sponge used had a size of 5x7, rIFG could have been affected by the current.

***Response: **This is a very good point that we have now addressed in the discussion. We based the placement of the electrodes on the study of Loo et al. (2012), who initially wanted to study the anti-depressant effect of tDCS but found an improvement of attention and working memory. Therefore, the electrode placement might not be optimal for working memory and attention. We have added this to the limitations section of the discussion.*

*“* *Fourth, the placement of the electrodes might not have been optimal to target attention deficits. Our study was based on the outcome of Loo et al. (2012) who aimed to investigate the anti-depressant effect in patients with depression, but found an improvement of attention and working memory instead (Loo et al., 2012). By copying this electrode placement, we hoped to replicate these results in patients with burnout. However, by placing the cathode on F8, we might have unwantedly inhibited the right inferior frontal gyrus, which has been linked to inhibition and attentional control (Hampshire et al., 2010). Although cathodal tDCS over the right inferior frontal gyrus did not appear to have a significant effect on response stopping or reaction times in a stop-signal task (Stramaccia et al., 2015), another choice for the cathodal reference electrode might be warranted.* *In addition, AF3 targets primarily the more frontal site of the left DLPFC, while a more common placement to target DLPFC in attention studies is F3 (Coffman et al., 2014).”*

Only continuous outcome measures were reported. Since it better reflects better clinical practice, I would add a categorical outcome measure, i.e., percentage of responders/remitters.

***Response: **Although we agree that, clinically, this would have added value, we do not really have a theoretical base for categorizing our participants into responders/remitters based on the outcome measures we used. Therefor we prefer to use the continuous scores for these outcome measures.*

A TCT device was used. The strap it comes with is made of a neoprene strap which could absorb the saline solution potentially increasing the surface area of the target area. Also, is this device EC approved?

***Response: **The TCT device is not EC approved but it was thoroughly tested in the lab of prof. dr. Mario Manto and approved by our ethical committee. The neoprene strap indeed can absorb the saline solution but during stimulation it was checked whether this absorption spread beyond the electrode surface. In addition, both electrodes were attached to the scalp using different straps to avoid bridges between the electrodes through the straps. This has now been added to the methodology:*

*“These were placed over the scalp using neoprene straps. Since these can absorb the saline solution, two different straps were used for both electrodes to avoid creating bridges and throughout the sessions the absorption was monitored so that it would not spread beyond the surface of the electrodes.”*

It was unclear whether all the patients in both conditions had been undergoing psychotherapy prior to the add-on tDCS therapy. If psychotherapy was new to all, it is a major confound. It could also be the reason why both groups improved burnout and depression-wise.

***Response: **Psychotherapy was new to all, therefore we did expect a significant improvement, burnout- and depression-wise, in both groups. We have added this also to the text. However, our main focus for the effect of tDCS was on the attention component, since personal experience learned that attention deficits are the most resilient symptom of burnout.*

*Methodology: “* *None of the patients had received psychotherapy before inclusion in this study.”*

Discussion: *“Both groups improved on all these measures **, which can be expected due to the behavioral therapy both groups received,** but the improvement of burnout and overall quality of life was only significant after real tDCS.”*

In the tDCS protocol section, Fp3 is mentioned as the target site, “left DLPFC”. Since the 10-20 system of electrode placement is used, defining the target site as AF3 is probably better than Fp3. Also, the vast majority of TMS/tDCS studies targeting left DLPFC to stimulate F3. Why was Fp3/AF3 selected? DLPFC is indeed a large area. But an elaboration on the reason why a more anterior part of DLPFC was chosen should be explained.

***Response: **As mentioned above, we based the placement of the electrodes on the study of Loo et al. (2012), who initially wanted to study the anti-depressant effect of tDCS. Therefore, this might indeed not be the ideal stimulation site for our purposes, but it has been proven to be effective in Loo et al. (2012). This has been added in the limitations section. We also changed Fp3 to AF3 as suggested.*

*“In addition, AF3 targets primarily the more frontal site of the left DLPFC, while a more common placement to target DLPFC in attention studies is F3 (Coffman et al., 2014).”*

**Minor Comments:**

In the background section of the abstract/methods, only one objective (attention and executive control) is mainly elaborated upon. I would also add the clinical objective, i.e., burnout, depression amelioration. Only QoL was briefly mentioned.

***Response: **Since we did expect a good outcome on burnout and depression scores with the behavioral therapy, the main focus of the study was on the added value of tDCS in improving the more resilient attention deficits. Therefore, we primarily elaborated on that objective. We hope that we have made this clearer as follows in the abstract:*

*Abstract: “Background: Burnout is characterized by deficiencies in attention and several components of the working memory **. It has been shown that cognitive behavioral therapy can have a positive effect on burnout and depressive symptoms, however, **the lingering effects of impaired attention and executive functions are the most frustrating.”*

The tDCS section of the introduction is not well structured. It’s better to subdivide the use of tDCS into neurological and psychiatric conditions. Now a distinction is made between “motor, cognitive, affective disorders” and “Alzheimer’s and MDD”.

***Response: **Thank you for the thorough reading of the manuscript, this was indeed badly phrased. We have now changed it into the following, hopefully better, sentence:*

*“tDCS is increasingly used in the treatment of motor, cognitive, and affective symptoms in different patient populations, **both in neurological (e.g. Alzheimer’s disease; Flöel, 2014), and psychiatric disorders (e.g. major depressive disorder; Nitsche et al., 2009) (Brunoni et al., 2012)**.”*

Two papers on depression are mentioned: one submitting patients to 1 session and the other to 15 sessions. I would talk about the importance of the number of sessions as a potential variable that could increase tDCS effect (Brunoni) ^2^ to explain why you submitted the patients to 15 sessions. Also, there are maybe one or two articles on stress in professionals, indirectly referring to burnout with no tDCS effect on burnout. One of the contributions of this article is that it may be the first looking at burnout. I would highlight this.

***Response: **In the introduction, we have added an explanation for using repeated sessions instead of a single session in our study and we have pointed out that our study is probably the first to study the effects of tDCS in a burnout population. Thank you for this suggestion!*

*“However, to induce a longer-lasting effect, repeated sessions are advised and it has already been shown that this can have a cumulative effect which is associated with greater magnitude and longer duration of the behavioral effects (Brunoni et al., 2012).*

*The effects of tDCS have not yet been extensively evaluated in burnout patients. Some studies have used tDCS in stress-related patient populations, such as professional nurses (Stanton et al., 2015) or post-traumatic stress disorder (Saunders et al., 2015), however, to our knowledge, our study is the first to use tDCS in a burnout population.”*

I would also go more into details of other potential brain regions that could have been targeted, especially for those tasks that did not show a tDCS effect. Only DLPFC is mentioned. E.g. as mentioned above, inhibition has not been linked to DLPFC. Or why you included those tasks while targeting DLPFC. It could be the reason why no significance was observed in inhibition.

***Response: **We have added several sentences to the discussion to clarify when we expected a result of left DLPFC stimulation and when it was rather unexpected. The discussion now reads as follows:*

*“This study showed that three weeks of therapy, combined with real or sham stimulation, significantly improved several components of the central executive. However, analyses revealed that improvement was driven by a significant improvement after real tDCS for inhibition and shifting (WCST), and was also primarily seen after real tDCS for updating and control (TMT A, D2 G z, D2 G z – F). These results are in line with the study of Miler et al. who found that atDCS over the left DLPFC significantly improves the executive control of attention ( Miler et al., 2018). As in the study of Miler et al., no effect was seen on the percentage of mistakes, but the processing speed did improve more on the D2 test of attention after real tDCS than after sham tDCS ( Miler et al., 2018). **However, the improvement on inhibition and shifting after tDCS was somewhat unexpected, since this is primarily associated with the right DLPFC and the right inferior frontal gyrus (Lie et al., 2006; Hampshire et al., 2010). Since this effect was only observed for the WCST, this might be related to this specific task. Indeed, studies have shown that, although the right DLPFC seems to be the most prominent in handling complex/manipulative working memory operations in the WCST (Lie et al., 2006), the left DLPFC is also involved during this task (Lie et al., 2006; Nagahama et al., 2005).***

*atDCS also seemed to have a positive impact on other components of the working memory. The phonological loop might also be positively influenced by real tDCS as shown by a significant improvement of the real tDCS group on the BNT, although the sham group also trended towards a significant improvement. No effect was seen on the visuospatial sketchpad **, which might not be surprising because this is believed to be situated primarily in the right prefrontal cortex (Suchan, 2008),** but encoding clearly improved more after real atDCS than after sham tDCS (RBANS Immediate Memory Index). **Transcranial magnetic stimulation (TMS) studies have shown a prominent role for the left DLPFC during encoding, observing shorter reaction times using a paired-pulse paradigm over this area (Gagnon et al., 2011). Though de Lara et al. (2017) did not find any effect of anodal tDCS over the left DLPFC on encoding, this might be due to the lesser intensity (1mA vs 2mA) they used in their study**.”*

A paragraph on neurotransmitters could also be added. Specifically, alterations in dopamine and noradrenaline levels in DLPFC have been associated with impaired working memory performance. TMS and tDCS over DLPFC have also been shown to release dopamine in various brain regions, including the right ventral striatum. Further, tDCS to DLPFC has shown to improve participants’ memory accuracy, an effect that has been correlated significantly with dopamine release.

***Response: **This might indeed be relevant information for the interested reader. We have added the following paragraph in the introduction:*

**

*“One of the mechanisms that might be responsible for the cognitive problems in burnout patients is a dopaminergic dysfunction in the prefrontal cortex. It has been shown that dopamine in the prefrontal cortex plays a critical role in working memory and cognitive control (Polizzotto et al., 2020; Cools & D’Esposito, 2011) and that (chronic) stress can have a deteriorating effect on the dopaminergic system in this area (Mizoguchi et al., 2000). tDCS has been known to interact with dopaminergic systems (Polizzotto et al., 2020) and therefore tDCS over the DLPFC might be able to restore dopaminergic prefrontal cortex function.”*

*In addition, a paragraph was added to the limitations section with the findings of a very recent paper on DLPFC stimulation and working memory:*

*“Lastly, research has shown that the effect of tDCS on working memory might be dependent on, amongst others, the initial dopaminergic level that can impact the excitation/inhibition balance (i.e. homeostasis between relative contributions of excitatory and inhibitory synaptic inputs) (Polizzotti et al, 2020). More insight into the exact working mechanisms underlying the cognitive and attention deficits in burnout patients might be beneficial for future research.”*

I would also mention briefly why it took 3 years to recruit 15 patients. It could be helpful for future studies recruiting patients with burnout.

***Response: **We have added some suggestions in the Limitations section to help with recruiting larger patient groups. As a single site with one therapist, it was very difficult to recruit several patients at the same time due to time constraints and a limited number of new intakes. In addition, several patients were discouraged by their primary physicians to participate in the experiment, since the primary physicians did not have enough information to judge the safety of tDCS.*

*“Our study has several important limitations. First, our group of patients was relatively small. This is an important limitation given the positive trends of the effect of real tDCS on several outcome measures. Studies with more power will have to show whether these trends failed to reach significance due to a lack of power. In addition, some variables of interest (D2 s-score, TMT B, Stroop Card II, Raven) could not be interpreted correctly with the linear mixed model analysis because of a violation of assumptions. More data points could help to resolve this issue. **Setting up multi-site cooperations to recruit participants and maintaining close relationships with primary care providers making them aware of the safety of tDCS when applied in the correct manner, could also help to convince patients to participate in tDCS studies. Larger groups to validate the efficacy of tDCS are crucial to investigate the clinical usability of this therapeutic aid**.”*

Were the patients asked at the end of the treatment whether they knew they had received sham or real? (chi-squared test to test the integrity of masking).

Were patients asked to evaluate their level of discomfort? The level of discomfort/pain has been associated with a TMS/tDCS effect.

***Response: **Unfortunately, we did not systematically address these issues. We have added this to the limitations section and recommended it for future studies.*

*“In addition, it is recommended to test for the efficiency of blinding the type of stimulation by asking the participants afterwards whether they think they were actively stimulated or not. Gathering information about the amount of discomfort could also be of importance for future studies using tDCS.”*
